# Recurrence–Preventive Role of Flatus Tubes Following Endoscopic Decompression in Sigmoid Volvulus

**DOI:** 10.5152/tjg.2023.22201

**Published:** 2023-04-01

**Authors:** Sabri Selçuk Atamanalp, Esra Dişçi, Rıfat Peksöz, Ercan Korkut, Nurhak Aksungur, Necip Altundaş, Salih Kara

**Affiliations:** 1 Department of General Surgery, Faculty of Medicine, Ataturk University, Erzurum, Turkey

**Keywords:** Endoscopic decompression, early recurrence, flatus tube, sigmoid volvulus

## Abstract

**Background:**

Sigmoid volvulus may recur following endoscopic decompression. Flatus tubes are traditionally used to prevent an early recurrence. This study aims to evaluate the recurrence–preventive role of the flatus tubes in sigmoid volvulus.

**Methods:**

Sigmoid volvulus recurrence was retrospectively analyzed in prospectively collected clinical data of endoscopically decompressed 60 patients, in whom no tube, rectal tube, or sigmoidal tube was used.

**Results:**

Mean pain/discomfort scores were higher in rectal and sigmoidal tube groups than that of no tube group (1.2 ± 0.4, 4.2 ± 0.9, and 3.5 ± 0.9, respectively, *P*  < .001). The early recurrence was seen in 3 patients in the no tube group, while no early recurrence was determined during tube placement in the rectal and sigmoidal tube groups (15.0%, 0.0%, and 0.0%, respectively, *P*  < .05, *P*  < .05, and *P*  > .05). The tubes were removed or spontaneously discharged in 13 (65.0%) and 12 patients (60.0%) in the rectal and sigmoidal tube groups, respectively, and sigmoid volvulus recurred in 2 patients in each group following the removal or spontaneous discharge. There was no statistically significant difference between the early recurrence rates of the no tube, rectal tube, and sigmoidal tube groups following the removal or spontaneous discharge of the tubes (15.0%, 15.4%, 16.7%, respectively, *P*  > .05) and in total (15.0%, 10.0%, and 10.0%, respectively, *P*  > .05).

**Conclusion:**

Flatus tubes may prevent the early volvulus recurrence during their placement in sigmoid volvulus. Nevertheless, they generally cause pain and discomfort, and they are frequently removed or spontaneously discharged, which suppresses their recurrence–preventive effects.

**Main Points**

- Sigmoid volvulus is a rare closed-loop colonic obstruction.
- Although endoscopic decompression is the first treatment option, the disease tends to recur.
- Despite inadequate randomized controlled data, a flatus tube is traditionally placed in the rectum or sigmoid colon to prevent recurrence.
- The flatus tubes may prevent or reduce the early volvulus recurrence during their placement. However, most of them are removed by the patients or the practitioners or discharged spontaneously, and the early recurrence rate may increase, which decreases their recurrence–preventive effect.
- The early recurrence in patients under medical observation may be treated with emergency surgery as a semi-elective procedure or as an alternative, elective surgery or percutaneous endoscopic colopexy following the repetitive endoscopic decompressions may be preferred.

## Introduction

Sigmoid volvulus (SV), the rotation of the sigmoid colon around its mesentery causing intestinal obstruction, recurs in 3%-86% of cases following successful endoscopic decompression.^1-3^ Although late recurrence may be reduced by elective sigmoid colectomy in selected patients,^3,4^ the prevention of early recurrence, which generally occurs in the first few hours during the index hospitalization period, is not that easy. For this reason, despite inadequate randomized controlled data,^5,6^ some clinicians traditionally place a flatus tube in the rectum or sigmoid colon for 1-3 days to prevent recurrence.^7-9^ Nevertheless, flatus tube placement is still based on low-quality evidence in current coloproctology and gastroenterology guidelines.^10-12^

Although providing a basis for further decompression and bowel preparation are other potential benefits of the flatus tube placement, its main purpose is to reduce the early recurrence in SV.^10,11^ However, since the first description of SV by Rokitansky in 1836,^13^ no paper depending on high-quality evidence was reported in the literature on this subject.^5,6^ The aim of this study is to evaluate the actual role of the flatus tube placement in SV in the light of our 55.5-year (from June 1966 to January 2022) and 1046-case experience, which is the largest single-center SV series in the world.^5,6^

## Materials and Methods

In this study, SV recurrence was retrospectively analyzed in clinical data of 60 patients with SV, in whom the data were collected prospectively. As a traditional clinical practice of our clinic, no flatus tube, rectal tube, or sigmoidal tube was placed following successful endoscopic decompression in a 9-year period between January 2013 and January 2022.

**Figure 1.**
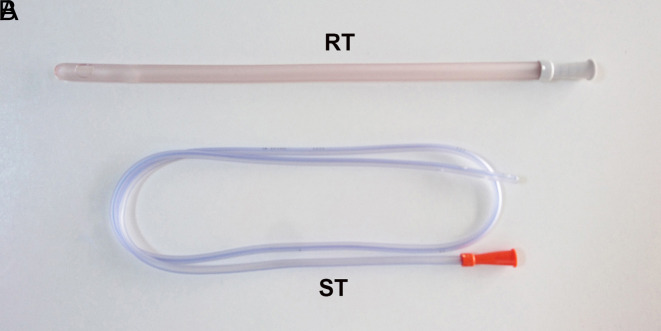
Flatus tubes (RT, rectal tube; ST, sigmoidal tube).

All procedures were performed by or under the supervision of a general surgeon. Patients with bowel gangrene or peritoneal irritation, or unsuccessful endoscopic decompression, who required emergency surgery, or those with American Society of Anesthesiologists (ASA) > 3 scores, were excluded from the protocol of the study. Similarly, patients, in whom clinical follow-up was impossible during the medical observation, were also excluded. In the endoscopic decompression, flexible sigmoidoscopes or colonoscopes (length: 70-170 cm, diameter: 1.2-1.5 cm) were used and when needed, propofol (2.5 mg/kg, intravenous [iv]) was used as premedication. In patients with evacuation of gas and stool, as well as relaxation of the abdomen, the endoscopic decompression was accepted as successful. According to flatus tube application, 60 patients were evaluated in 3 groups each involving 20 consecutive cases. In “no tube group,” no flatus tube was used, while in the “rectal tube group,” a rectal tube (length: 25-30 cm, diameter: 1-1.5 cm, Figure 1A) was placed in the rectum next to the endoscope under the endoscopic guidance before the removal of the instrument, and in the “sigmoidal tube group,” a nasogastric tube (length: 50-70 cm, diameter: 0.8-1.2 cm, Figure 1B) was inserted throughout the sigmoid colon under the guidance of a guidewire placed by the working channel of the instrument and following its removal. All tubes were fixated onto the buttocks by an adhesive tape and they were removed following a 24-hour observation period, while they were not blindly replaced in cases with removal by the patients or practitioners, or spontaneous discharge. Patients with early recurrence were treated with emergency surgery as a semi-elective procedure. In non-recurrent patients with ASA scores 1-3, elective surgery was suggested and performed in approvers, while other cases were discharged 24-48 hours later. During this period, the reappearance of SV symptoms including abdominal pain, distention, and obstipation associated with radiological findings including coffee-bean sign or mesenteric whirl sign was accepted as early recurrence criteria.

For each patient, age, gender, previous volvulus attacks, ASA score, pain/discomfort score, removal or spontaneous discharge, early recurrence (within 24 hours in index hospitalization period), morbidity, and mortality were noted. The pain/discomfort scores were evaluated as; 1: no, 2: minimal, 3: moderate, 4: severe, and 5: intolerable pain and/or discomfort.

Informed consent was obtained from all patients. This study was approved by institutional review board (Ethical Committee of Ataturk University Faculty of Medicine, B.30.2.ATA.0.01.00/88-2022).

### Statistical Analysis

Statistical analysis was performed using Statistical Package for the Social Sciences version 22.0 software (IBM, Armonk, NY, USA). Data were expressed as mean ± standard deviation or median (minimum-maximum) for numerical variables, while as numbers and percentages for categories. The normality distribution of the univariate data was examined with the Shapiro–Wilk Francia test, while the Levene test was used in variance homogeneity. In the evaluation of triple groups, as a parametric test, one-way analysis of variance test, and as a nonparametric test, Kruskal–Wallis test Monte Carlo simulation technique was used, while post hoc analysis was performed by Dunn’s test. In the evaluation of categorical variables, the Pearson chi-square test Monte Carlo simulation technique was used. Data were analyzed at a 95% CI and statistical significance was set at *P*  < .05.

## Results

During a 9-year period between January 2013 and January 2022, the endoscopic decompression was tried in 80 (85.1%) of 94 patients with SV and it was successful in 70 patients (87.5%). Among the 60 patients included in the study, the mean age was 64.1 years (range: 35-75), and 49 of them (81.7%) were male.

The flatus tube placement and related results are summarized in Table 1. As seen, there was no statistically significant difference between the preoperational parameters including the mean ages (62.5 ± 10.5, 61.6 ± 11.1, and 60.9 ± 8.8 years, respectively, *P*  > .05), male/female ratios (16/4, 17/3, and 16/4, respectively, *P*  > .05), mean previous volvulus attacks (0.3 ± 0.6, 0.3 ± 0.5, and 0.3 ± 0.7, respectively, *P*  > .05), and mean ASA scores (2.2 ± 0.8, 2.3 ± 0.7, and 2.1 ± 0.9, respectively, *P*  > .05) of the patients in the no tube, rectal tube, and sigmoidal tube groups.

During or following the tube application procedures, 19 patients (95.0%) in the rectal tube group and 17 cases (85.0%) in the sigmoidal tube group complained about the flatus tube. The mean pain/discomfort scores were significantly higher in the rectal tube and sigmoidal tube groups than that of the no tube group, while there was no statistically significant difference between the rectal tube and sigmoidal tube groups (1.2 ± 0.4, 4.2 ± 0.9, and 3.5 ± 0.9, respectively, *P*  < .001, *P*  < .001, and *P*  > .05).

In this series, the early recurrence was seen in a total of 7 patients (11.7%), 3 of them in the no tube group, while no early recurrence was determined as long as tube placement in 7 patients in the rectal tube group and in 8 patients in the sigmoidal tube group. In this way, both kind of flatus tubes statistically prevented the early SV recurrence during their placement (15.0%, 0.0%, and 0.0%, respectively, *P*  < .05, *P*  < .05, and *P*  > .05). However, the flatus tubes were removed or spontaneously discharged in 13 patients (65.0%) in a mean 10.5-hour period (range: 5 minutes-16 hours) and in 12 patients (60.0%) in a 12.1-hour period (range: 30 minutes-18 hours) in the rectal and sigmoidal tube groups, respectively, and SV recurred in 2 patients in each group (15.4% and 16.7%, respectively) following the removal or discharge of the flatus tubes. In this way, total early recurrence rates were established as 10.0% in each of the latter groups. There was no statistically significant difference between the early recurrence rates of the no tube, rectal tube, and sigmoidal tube groups following the removal or spontaneous discharge of the tubes (15.0%, 15.4%, 16.7%, respectively, *P*  > .05) and in total (15.0%, 10.0%, and 10.0%, respectively, *P*  > .05).

In this series, 7 patients (11.7%) with early recurrence were treated with the emergency surgery as a semi-elective procedure including open sigmoid colectomy and primary anastomosis. No major complication was determined in patients treated with endoscopic decompression, while acute anal fissure occurred in 2 patients (10.0%) and 1 patient (5.0%) in the rectal and sigmoidal tube groups, respectively. On the other hand, incision site infection was seen in 2 patients (28.6%) in surgically treated 7 patients. No mortality was determined in this series.

## Discussion

Based on the variable-based clinical evidence, current guidelines including the American Society for Gastrointestinal Endoscopy 2020 guideline,^10^ the American Society of Colon and Rectal Surgeons 2021 guideline,^11^ and the Association of Coloproctology of Great Britain and Ireland 2021 guideline^12^ strongly recommend the endoscopic decompression as the first choice in patients without peritonitis, bowel gangrene, and perforation in the management of SV. However, the flatus tube placement following successful endoscopic decompression is still a controversial subject and is still based on low-quality evidence.^10-12^

According to the limited data obtained from the flatus tube- and early recurrence-related articles indexed in Web of Science^5^ and PubMed^6^ databases in the last 53 years (from 1970 to date), the placement of the flatus tubes following the successful endoscopic decompression in SV series and their recurrence–preventive roles are summarized in Table 2.^2,7-9,14-35^ In consideration of the present data, all of which depend on retrospective archive research except for a few uncontrolled prospective reports, the early recurrence rates may rise to 66.7% in patients with flatus tubes, while 50.0% in the others. Due to the absence of a prospective controlled clinical study and the rarity of the reports on this subject from the first description of SV in 1836 to date,^5,6,13^ obtaining a healthy result is not easy. Nevertheless, about 40% of the practitioners traditionally apply flatus tubes to prevent the early recurrence of SV.^24^ In our opinion and experiment, other causes of this practice may be the practitioners’ achievement of personal peace and comfort, as well as their avoidance of probable medicolegal responsibilities arising from the early SV recurrence, which is a headache for both clinicians and patients.

No matter how useful it is, from our clinical experience, flatus tube placement is a relatively painful and uncomforting procedure in its application, retaining, and removing. Additionally, most of the flatus tubes are spontaneously discharged in the course of the first defecation or degasification, even if on walking or in resting. In addition to the spontaneous discharge, some patients remove the flatus tubes due to the pain and discomfort, while some others obligate the practitioners to remove them. The relatively high pain/discomfort scores due to the usage of the flatus tubes, as well as the relatively high flatus tube removal or discharge rates in the present study, support this idea. Once the flatus tubes are discharged or removed, repetitive applications are generally neglected by both the patients and the practitioners. Even if reapplication is possible, blind replacement is generally preferred instead of repetitive endoscopic manipulation, which may court complications.^36^ Although some contrariwise studies demonstrate relatively high early recurrence rates,^9,22,27,30,31^ the flatus tubes may prevent or reduce the early recurrence during their placement period in SV,^8,15,20,35,36^ as was in our study. However, the present study demonstrates that, following the removal or spontaneous discharge, their recurrence–preventive role, which is the main purpose of their usage, may decrease and the early recurrence rate may come up to similar levels.

In the flatus tube application in SV, short rectal tubes are the most preferred instruments, particularly due to their easy implementation practice.^8,14,15,17,18,20,25,27,28,31,33^ However, long sigmoidal or nasogastric tubes are the less-favored apparatus arising from their practical difficulty.^7,9,15,30,33^ Although the results of this study did not demonstrate any statistically significant difference between the pain/discomfort scores of the 2 types of flatus tubes, according to our clinical experiment, sigmoidal or nasogastric tubes are relatively better tolerated by the patients arising from smaller diameters and more flexible bodies, which hurt less.

The flatus tubes may be placed by different techniques. Although blind placement is an easy and quick procedure, it is not free from complications including bowel perforation.^37^ As the most frequently used method, the flatus tubes may be placed within the rigid endoscope, while next to the rigid or flexible endoscopes before their removal, alternatively, they are placed under the guidance of a guidewire placed by the working channel of the flexible endoscope and following its removal.^7,9,27,33^ In the lassoing technique, the head of the flexible endoscope is hooked by a snare wire placed by the working channel of the instrument and inserted together with it.^38^ As an alternative, a pediatric flexible endoscope (diameter: 5-6 cm) may be placed through the lumen of a large flatus tube (24 F) and may be placed as a single combined tube.^39^ Finally, fluoroscopic guidance may be used.^40^ In our opinion, the basic rule of flatus tube placement is to use endoscopic or fluoroscopic guidance to avoid complications. The flatus tubes are generally fixated onto the buttocks by adhesive tape.^9,33^

Except for the bowel perforation and related death risk, which is a rare outcome of the blind replacement, no major complication or mortality is reported corresponding with the flatus tube placement.^37^ Retention of the flatus tube is another rare complication, which requires repetitive endoscopic examinations and removal.^41^

Regarding the treatment options for early SV recurrence, although every volvulus attack carries some risks of morbidity and mortality,^3^ in our experience, early and correct SV diagnosis is generally possible in such patients under medical observation. Additionally, this observation period allows for an improvement in the general condition of the patients arising from the treatment of the comorbidities, antibiotic prophylaxis, and bowel cleansing. For this reason, we prefer emergency sigmoid colectomy and primary anastomosis as a semi-elective procedure with acceptable morbidity and mortality rates in patients with early recurrence. However, a rationalist alternative may be the repetitive endoscopic decompressions followed by elective surgery within 2-5 days in patients with ASA score 1-3, while elective percutaneous endoscopic colopexy (PEC) in cases with ASA score ≥4.^13,32^

As a result, although the number of the subjects is the major limitation, to evaluate 60 cases with SV, a 9-year clinical effort was required in the present study. It is clear that to obtain an outlasting study, it may get a few 10 years, which may make the results questionable due to improving opportunities in time. Additionally, the retrospective evaluation of the present study may be thought of as another drawback or shortcoming, whereas a prospective controlled clinical study may give more credible results about the recurrence–preventive role of the flatus tubes in SV.

In conclusion, the flatus tubes may prevent or reduce early SV recurrence during their placement. However, most of them are removed by the patients or the practitioners arising from the achiness and unsetting nature of the procedure or spontaneous discharge. Following the removal or spontaneous discharge, their recurrence–preventive effect may disappear and the early recurrence rate may increase resulting in similar clinical evidence. The early recurrence in patients under medical observation may be treated with emergency surgery as a semi-elective procedure or as an alternative, elective surgery or PEC following the repetitive endoscopic decompressions may be preferred.

## Figures and Tables

**Table 1.** The Parameters and the Statistical Analyses

Parameter/Group	No Tube	Rectal Tube	Sigmoidal Tube	Statistical Analysis
Age (mean ± SD, range)	62.5 ± 10.5 (35-75)	61.6 ± 11.1 (38-74)	60.9 ± 8.8 (36-73)	.566
Gender (male/female)	16/4 (80.0%/20.0%)	17/3 (85.0%/15.0%)	16/4 (80.0%/20.0%)	.895
Previous volvulus attack (mean ± SD, case)	0.3 ± 0.6 5 (25.0%)	0.3 ± 0.5 6 (30.0%)	0.3 ± 0.7 5 (25.0%)	.538
ASA score (mean ± SD, 1/2/3)	2.2 ± 0.8 4/8/8	2.3 ± 0.7 3/8/9	2.1 ± 0.9 6/6/8	.823
Pain/discomfort score (mean ± SD, 1/2/3/4/5)	1.2 ± 0.4 (17/3/0/0/0)	4.2 ± 0.9 (0/1/3/8/8)	3.5 ± 0.9 (0/3/7/7/3)	< .001*
Removal/spontaneous discharge	-	13/20 (65.0%)	12/20 (60.0%)	.747
Early recurrence (with tube)	3/20 (15.0%)	0/7 (0.0%)	0/8 (0.0%)	.042*
Early recurrence (without tube)	3/20 (15.0%)	2/13 (15.4%)	2/12 (16.7%)	.992
Early recurrence (total)	3/20 (15.0%)	2/20 (10.0%)	2/20 (10.0%)	.851

*No tube—rectal tube and no tube—sigmoidal tube: significant, rectal tube—sigmoidal tube: nonsignificant.

**Table 2.** Data on Flatus Tube Placement Following Successful Endoscopic Decompression and Early Recurrence in Sigmoid Volvulus

Author	Year	Design	Flatus Tube	Patient	Early Recurrence	Late Recurrence
Arnold and Nance^14^	1973	RE	RT	55	?	30 (54.5%)
Starling^15^	1979	PR, UC	RT, ST	3	0 (0.0%)	?
Ballantyne et al^16^	1985	RE	?	14	0 (0.0%)	?
Bak and Boley^17^	1986	RE	RT	33	?	20 (60.6%)
Brothers et al^18^	1987	RE	RT	16	?	9 (56.3%)
Bhuiyan et al^19^	2005	RE	?	10	0 (0.0%)	?
Heis et al^20^	2008	RE	RT	15	0 (0.0%)	?
Tan et al^21^	2010	RE	RT	52	?	28 (53.8%)
Mulas et al^22^	2010	RE	RT	29	13 (44.8%)	?
Codina Cazador et al^23^	2011	RE	RT, NT	29	?	18 (62.1%)
Swenson et al^24^	2012	RE	RT, NT	19	?	10 (47.6%)
Yassaie et al^25^	2013	PR, UC	RT, NT	31	?	19 (61.3%)
Lou et al^26^	2013	RE	?	26	7 (26.9%)	?
Maddah et al^27^	2014	RE	RT	28	6 (21.4%)	?
Ifversen and Kjaer^28^	2014	RE	RT, NT	26		14 (53.8%)
Sugimoto et al^29^	2014	RE	NT	18		10 (55.6%)
Bruzzi et al^30^	2015	RE	ST	33	22 (66.7%)	?
Colinet et al^31^	2015	RE	RT, NT	12	6 (50.0%)	?
Iida et al^32^	2017	RE	?	13	6 (46.2%)	?
Johansson et al^2^	2018	RE	?	11	4 (36.4%)	?
Atamanalp^33^	2019	PR, RE	RT, ST, NT	560	28 (5.0%)	?
Quénéhervé et al^7^	2019	RE	ST	74	?	25 (33.8%)
Kim et al^34^	2020	RE	?	39	6 (15.4%)	?
Firat et al^35^	2020	RE	NT	18	0 (0.0%)	?
Gonzalez-Urquijo et al^8^	2020	RE	RT	8	0 (0.0%)	?
Surek et al^9^	2021	RE	NGT	52	18 (34.6%)	?

RE, retrospective; PR, prospective; UC, uncontrolled; RT, rectal tube; ST, sigmoidal tube; NGT, nasogastric tube; NT, no tube; ?, No information.
